# Impact of genomic selection for disease resistance on the spread of infection in a simulated aquaculture population

**DOI:** 10.1186/s12711-026-01072-7

**Published:** 2026-09-07

**Authors:** Silvia García-Ballesteros, Jesús Fernández, Andrea B. Doeschl-Wilson, Beatriz Villanueva

**Affiliations:** 1 Departamento de Mejora Genética Animal, INIA-CSIC, 28040 Madrid, Spain; 2 The Roslin Institute, University of Edinburgh, Easter Bush, Edinburgh, EH25 9RG UK

## Abstract

**Background:**

In aquaculture, selection for disease resistance is typically based on mortality records from challenge tests performed on relatives of selection candidates. However, commercial success depends on limiting disease transmission, particularly the incidence and severity of outbreaks. It remains unclear whether selecting for lower mortality also reduces disease transmission. Both these outcomes are influenced by three underlying epidemiological host traits: susceptibility, infectivity, and infection-induced mortality. This simulation study evaluated the impact of genomic selection against mortality on disease transmission in a salmon population exposed to a pathogen with a fast transmission rate.

**Methods:**

Mortality was assumed to be recorded on sibs of selection candidate, either as binary dead/alive status or as time to death during cohabitation/bath challenge tests. Phenotypes were simulated using a stochastic compartmental Susceptible-Infected-Removed epidemiological model, with genetic variation for the three underlying traits. Scenarios were explored by varying the genetic correlations between the three underlying traits. Challenge test designs varied in the number of groups, group sizes, and family distribution across groups. For comparison, a reference scenario with direct selection on the underlying traits was included. Genomic selection was applied over 10 discrete generations, and its impact on disease transmission was assessed using the basic reproductive ratio (R_0_).

**Results:**

When selection was based on dead/alive status, R_0_ was highly sensitive to both the genetic correlations between the three underlying traits and the challenge test design. In contrast, selection based on time to death consistently reduced R_0_ across all scenarios (often to below 1 within four generations), regardless of trait correlations or test design. Selection on time to death primarily produced fish with reduced susceptibility to infection, while selection on dead/alive status produced fish with increased resistance and endurance to infection, delaying onset of infection or death without necessarily limiting transmission. Direct selection on the underlying epidemiological traits was the most efficient approach to reduce both mortality and transmission.

**Conclusions:**

Genomic selection for disease resistance, when measured as time to death in cohabitation or bath challenge tests conducted until mortality naturally levels off, reduces both mortality and disease spread. Breeding programs may benefit from challenge test designs that enable estimation of genetic parameters for the underlying traits affecting disease transmission and survival.

## Background

Infectious disease outbreaks are a major threat to the economic and environmental sustainability of animal production industries [1–3]. This threat is particularly pronounced in aquaculture, where the specific production conditions, such as the sharing of a common environment in tanks or cages and the water flow, facilitate transmission of infectious diseases and increase the difficulty of controlling them beyond conditions for terrestrial species. While appropriate management plans and vaccination are crucial, selective breeding can also play a critical role in preventing outbreaks [e.g., 4].

In aquaculture species with advanced selective breeding programs, such as salmonids and shrimp, disease resistance is already included as a breeding objective [5–9]. In recent years, there has also been a significant increase in studies aimed at estimating genetic parameters for resistance to important diseases in other species, with the general aim of determining the potential of selection for this trait [see reviews of 10, 11].

Selection for disease resistance in aquaculture typically focuses on enhancing individual resilience to infection, specifically, improving a fish’s ability to survive pathogen exposure and thereby reducing mortality rates. However, to maximize the effectiveness and commercial success of selective breeding programs, the focus should extend beyond mortality reduction to also include limiting disease transmission by reducing the incidence and severity of outbreaks in the population [12]. Breeding fish that survive infection but that remain highly infectious and shed large quantities of pathogens has limited benefits. Therefore, combining genetic and epidemiological models is essential for achieving more effective and sustainable disease control.

Numerous studies have emphasized the significance of the basic reproductive ratio (R_0_) in regulating infectious disease transmission and its dependence on the three epidemiological host traits that are under potential genetic control, including susceptibility, infectivity, and infection-induced mortality [12–14]. R_0_ is defined as the expected number of secondary infections that one infectious individual causes in an otherwise susceptible population [15–17]. Susceptibility is defined as the propensity of an uninfected individual to become infected when exposed to infectious material. Infectivity is defined as the ability of an individual, once infected, to transmit the infection. Finally, infection-induced mortality is defined as the propensity of an infected individual to die. Note that, when no alternative outcomes exist (e.g., recovery, withdrawal, censorship), infection-induced mortality is effectively the inverse of endurance, as described in previous aquaculture studies [18–20], which is the propensity of an infected individual to survive the infection. From an epidemiological perspective, an optimal host population would consist of individuals with low susceptibility, low infectivity, and high infection-infection mortality (i.e., infected animals are rapidly removed after infection). Although direct selection for the underlying epidemiological traits could be key in reducing infectious disease transmission, unfortunately, these traits are not readily observable.

In aquaculture breeding programs, disease resistance is typically assessed through mortality, recorded either as a continuous (time to death) or a binary (dead or alive status) trait in challenge tests [5, 9, 19, 21]. Depending on the specific aims, the pathogen in question, and other factors, infection challenges can be administered through inoculation, injection, bath exposure (where a specific pathogen dose is added to the water), or cohabitation with infected seeder individuals. In practice, challenge tests often involve a limited number of large groups with families evenly mixed, mainly due to constraints in tank space [20, 22–24]. However, accurate estimation of genetic infectivity requires test designs that allow natural pathogen transmission (i.e., bath or cohabitation challenge) and the use of many small epidemiological groups [25, 26]. Both types of tests involve exposing fish to a specific pathogen under controlled conditions at facilities that are physically separated from the breeding nucleus. As a result, phenotypic data cannot be collected directly from selection candidates and are instead obtained from their sibs. This limitation prevents the exploitation of within-family genetic variation when using traditional BLUP selection. However, the advent of genomic selection has proven particularly beneficial for such sib traits, as it enables the capture of both between- and within family genetic variation, thereby significantly enhancing selection accuracy [8, 9]. Selecting for the observable traits, such as time to death or dead/alive status, likely significantly impacts the underlying traits and, thus, disease transmission. Mortality in an individual depends on its own susceptibility, the infectivity of other fish in the group, and its own infection-induced mortality. However, to what extent focusing solely on mortality without taking into account the epidemiology of the disease would decrease pathogen transmission and disease spread remains unclear [12, 20, 27].

The aim of this study was to predict the effects of implementing genomic selection against mortality on the spread of the disease, quantified by R_0_, as well as on the three epidemiological underlying traits. This was investigated using computer simulations that modeled an aquaculture population exposed to an infectious pathogen under challenge test conditions. Different genetic correlations between the underlying traits and different challenge test designs were evaluated.

## Methods

### Overview of the simulation process

The scenarios simulated involved genomic selection for 10 discrete generations, applied either (i) directly on the underlying traits; or (ii) against mortality, measured either as time to death (i.e., a continuous trait) or as dead/alive status at the end of the challenge test (i.e., a binary trait). The disease was assumed to be produced by a virus with a fast transmission rate. Specifically, the epidemiological parameters assumed were similar to those of the Infectious Salmon Anemia (ISA) virus, which is of significant concern in salmon production.

When selecting against mortality, records were obtained from sibs of the selection candidates in a simulated disease cohabitation challenge experiment. For time to death (number of days from the start of the challenge test until a fish’s death) recording, the phenotype was expressed as the sum of two components: the infection time (i.e., days from the start of the challenge until infection) and the infection-induced mortality time (i.e., days from infection until death). These times were obtained by implementing a stochastic compartmental Susceptible-Infected-Removed (SIR) epidemiological model.

The three underlying traits susceptibility, infectivity, and infection-induced mortality, that were incorporated into the SIR model to derive mortality phenotypes, were assumed to be polygenic. The initial phenotypic variance and heritability (*h*^2^) for each of these traits were 1 and 0.5, respectively. These parameter values led to a *h*^2^ of approximately 0.2 for the observed trait (mortality). Different genetic correlations between the underlying traits were considered. The R_0_ was then calculated as outlined below to assess the impact of selection for mortality on spread of the disease. A higher value of R_0_ indicates a greater risk and severity of epidemics, whereas a value of R_0_ below one indicates that the infection is unlikely to propagate and is expected to die out [e.g., 4, 14, 28]. The simulations were carried out with our own Fortran 90 codes.

### Simulation of the genome

The simulated genome for individuals in the population was composed of 30 chromosomes of 1 Morgan each, mirroring the salmon genome. A total of 3,600,000 biallelic loci with initial frequencies of 0.5 were simulated. Loci were evenly distributed across the genome.

### Base population

The base population (*t* = 0) from where the selection program started was created in a three-step process [29]. First, a population in mutation-drift equilibrium was generated by simulating 3000 discrete generations of random mating among 200 individuals (100 males and 100 females). Sires and dams were randomly sampled with replacement, and population size was kept constant across generations. The mutation rate per locus and generation was 2.5 × 10^–4^. Mutations were randomly distributed across individuals, chromosomes, and loci, switching allele 0 to allele 1 and vice versa. When generating the gametes, the total number of crossovers was drawn from a Poisson distribution with mean equal to the genome length in Morgans. Crossovers were randomly distributed without interference. At the end of this process, the expected heterozygosity of the population had already reached an equilibrium value (i.e., mutation-drift equilibrium).

In the second step, the population was expanded once in a single generation. One male and one female were randomly sampled with replacement from the population at equilibrium to produce one offspring from each mating, and repeating the process until a total of 500 males and 500 females were generated. These 1000 individuals constituted the expanded population.

Finally, in order to establish a base population with family structure, 100 males and 200 females were randomly sampled from the expanded population for each replicate. Each male was randomly mated to two females in a nested design, producing 30 offspring per mating. This resulted in a base population of 6000 fish per replicate, structured into 200 full-sib families and 100 paternal half-sib families.

At *t* = 0, a total of 48,000 loci with a minor allele frequency (MAF) ≥ 0.1 were randomly chosen across the genome for their use in genomic evaluation, resulting in a marker density of 1600 SNPs/Morgan. Additionally, from the full set of loci with MAF ≥ 0.1 (1,020,000 loci, including the 48,000 SNPs), 1000 were randomly selected as quantitative trait loci (QTLs) that simultaneously affected the three underlying polygenic traits (susceptibility, infectivity, and infection-induced mortality). To simulate specific genetic correlations between the traits, each QTL was assigned distinct effects on the three traits, as described in the following section. At *t* = 0, the average linkage disequilibrium, measured as the squared correlation between pairs of loci [30] was consistent with empirical observations in salmon. For instance, the average linkage disequilibrium between SNPs 50 kb apart was around 0.31, aligning with values reported by [31, 32].

### Underlying epidemiological traits

As previously mentioned, the three simulated underlying traits affecting disease transmission were susceptibility (*g*), infectivity (*f*), and infection-induced mortality (*r*). The true breeding value of individual *j* for trait *k* (TBV_*kj*_; *k* = *g*, *f*, *r*) was obtained as the sum of additive genetic values across QTLs. For QTL *i* affecting trait *k*, these values were *a*_*ik*_, 0 and − *a*_*ik*_ for homozygotes 11, heterozygotes 10 or 01, and homozygotes 00, respectively. The value ${a}_{{i}_{k}}$ was obtained in two steps [29, 33]. Firstly, a random number (${a}_{{i}_{k}}^{*}$) was sampled from the following multivariate normal distribution

$$N\left(\left[\begin{array}{c}0\\ 0\\ 0\end{array}\right],\left[\begin{array}{ccc}1& {\rho}_{{a}_{g,f}}& {\rho}_{{a}_{g,r}}\\ & 1& {\rho}_{{a}_{f,r}}\\ Symm& & 1\end{array}\right]\right),$$

where ${\rho}_{{a}_{k,l}}$ is the genetic correlation between traits *k* and *l* (*k* = *g*, *f*, *r*; *l* = *g*, *f*, *r*; *k* ≠ *l*). Secondly, ${a}_{{i}_{k}}$ was obtained by multiplying ${a}_{{i}_{k}}^{*}$ by the factor $\sqrt{\left\{{\sigma}_{{a}_{k}}^{2}/\left[2p(1-p){n}_{\mathrm{Q}\mathrm{T}\mathrm{L}}\right]\right\}}$, where ${\sigma}_{{a}_{k}}^{2}$ is the assumed additive variance of trait *k* at *t* = 0, *p* is the average frequency across QTLs at *t* = 0, and ${n}_{\mathrm{Q}\mathrm{T}\mathrm{L}}$ is the number of QTLs (i.e., 1000). Note that in this way the expected additive variance summed over all QTLs equals ${\sigma}_{{a}_{k}}^{2}$, assuming that covariances between loci generated by linkage disequilibrium are negligible. The phenotypic value of individual *j* for trait *k* was obtained by adding an environmental effect to TBV_*kj*_. This environmental effect was sampled from the following multivariate distribution

$$N\left(\left[\begin{array}{c}0\\ 0\\ 0\end{array}\right],\left[\begin{array}{ccc}{\sigma}_{{e}_{g}}^{2}& {\rho}_{{e}_{g,f}}{\sigma}_{{e}_{g}}{\sigma}_{{e}_{f}}& {\rho}_{{e}_{g,r}}{\sigma}_{{e}_{g}}{\sigma}_{{e}_{r}}\\ & {\sigma}_{{e}_{f}}^{2}& {\rho}_{{e}_{f,r}}{\sigma}_{{e}_{f}}{\sigma}_{{e}_{r}}\\ Symm& & {\sigma}_{{e}_{r}}^{2}\end{array}\right]\right)$$

where ${\sigma}_{{e}_{k}}^{2}$ is the environmental variance for trait *k* and ${\rho}_{{e}_{k,l}}$ is the environmental correlation between traits *k* and *l*. The environmental variance ${\sigma}_{{e}_{k}}^{2}$ was obtained as ${\sigma}_{{p}_{k}}^{2}-{\sigma}_{{a}_{k}}^{2}$, where ${\sigma}_{{p}_{k}}^{2}$ is the phenotypic variance for trait *k* that was set to one for the three traits. The environmental correlation was obtained as ${\rho}_{{e}_{k,l}}=({\rho}_{{P}_{k,l}}-{h}_{k}{h}_{l}{\rho}_{{a}_{k,l}})/ {e}_{k}{e}_{l}$, where ${h}_{k}, {h}_{l}$ are the square roots of the heritabilities for traits *k* and *l*, respectively, ${e}_{k}$ is $1-{h}_{k}^{2}$, ${e}_{l}$ is $1-{h}_{l}^{2}$, and ${\rho}_{{P}_{k,l}}$ is the phenotypic correlation between the two traits [35]. Note that at *t* = 0, the average TBV, and the average phenotypic value for the three underlying traits were zero.

Seven different scenarios varying in the genetic correlations assumed between the underlying traits were considered (Table 1). In scenario NC, the three traits were assumed to be uncorrelated at the genetic level. The FC scenarios represented favorable genetic correlations, from the perspective of reducing disease transmission. In scenario FC_*gf*_, a positive genetic correlation was assumed between susceptibility and infectivity. In scenarios FC_*fr*_ and FC_*gr*_, negative genetic correlations were assumed between infectivity and infection-induced mortality, and between susceptibility and infection-induced mortality, respectively.

**Table 1:** Genetic correlations between the underlying epidemiological traits assumed in the different scenarios

Scenario	${\rho}_{{a}_{g,f}}$	${\rho}_{{a}_{g,r}}$	${\rho}_{{a}_{f,r}}$
NC	0.0	0.0	0.0
FC_*gf*_	0.5	0.0	0.0
FC_*fr*_	0.0	0.0	− 0.5
FC_*gr*_	0.0	− 0.5	0.0
FC_*gfr*_	0.5	− 0.25	− 0.5
UC_*gf*_	− 0.5	0.0	0.0
UC_*fr*_	0.0	0.0	0.5
UC_*gr*_	0.0	0.5	0.0
UC_*gfr*_	− 0.5	0.25	0.5

${\rho}_{{a}_{g,f}}$: genetic correlation between susceptibility and infectivity; ${\rho}_{{a}_{g,r}}$: genetic correlation between susceptibility and infection-induced mortality; ${\rho}_{{a}_{f,r}}$: genetic correlation between infectivity and infection-induced mortality

Conversely, the UC scenarios represented unfavorable genetic correlations for transmission control. In scenario UC_*gf*_, susceptibility and infectivity were negatively correlated at the genetic level. In UC_*fr*_ and UC_*gr*_, positive genetic correlations were assumed between infectivity and infection-induced mortality, and between susceptibility and infection-induced mortality, respectively.

Importantly, note that correlations between traits were classified as favorable (scenarios FC) or unfavorable (scenarios UC) with respect to their impact on disease spread, not with respect to individual survival. For example, in scenario FC_*fr*_, fish with lower infectivity would also exhibit higher infection-induced mortality (i.e., lower endurance). Although rapid death from infection is detrimental to the individual, it limits the time available for transmission, making it beneficial from an epidemic control standpoint. Ideally, from this perspective, individuals should be the least susceptible, the least infectious, and die quickly upon infection.

Finally, two scenarios were included with genetic correlations between the three underlying traits to represent the extremes of genetic influence on transmission. The most favorable scenario (FC_*gfr*_) combined favorable genetic correlations between all underlying traits. In the same way, the least favorable scenario (UC_*gfr*_) included unfavorable genetic correlations. Note that in these scenarios, the third genetic correlation is constrained by the values of the other two and cannot reach high absolute values; accordingly, it was set to − 0.25 or 0.25.

### Challenge test

**Fig. 1:**
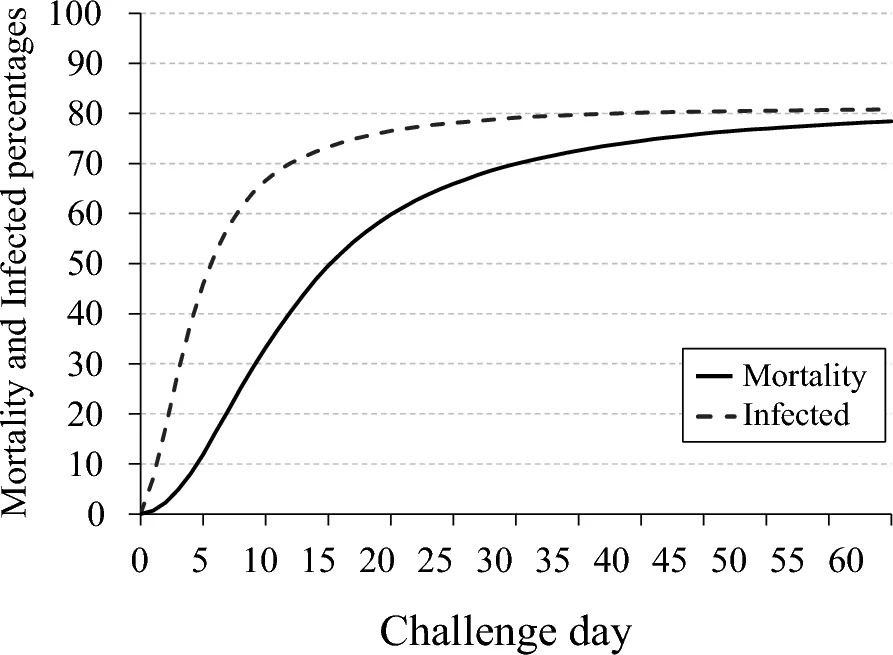
Cumulative mortality and infected percentages over 60 days during the simulated fast transmission challenge. This simulated curve was based on published data for infectious salmon anemia virus

Mortality phenotypes (time to death and dead/alive status) were obtained from the sibs (20 per family) of the selection candidates (10 per family) by simulating a disease cohabitation challenge experiment characterized by rapid transmission. These challenge tests were conducted experimentally for genetic evaluation purposes and are not intended to replicate natural epidemics in production farms. The epidemiological parameters assumed were based on published data for the ISA virus, as described in [20, 22, 36]. Following these studies, at *t* = 0, the simulated cumulative mortality reached approximately 80%, which was the asymptotic value achieved around day 50 from the start of the challenge test. Based on published cumulative mortality to ISA infection, the mean infection time and infection-induced mortality time were assumed to be 7 days (± 3 days) and 11 days (± 4 days), respectively, with a mean time to death of 18 days (± 7 days). Simulated cumulative mortality and infected percentages over time are shown in Fig. 1. The mean *per capita* transmission rate for the disease (*β*) and the mean *per capita* infection-induced death rate (*γ*) were calibrated by matching simulated infection and infection-induced mortality times to the cumulative mortality dynamics reported in experimental ISA challenge studies. The calibrated values of *β* and *γ* were 0.8 and 0.4 respectively, leading to an R_0_ ~4 at *t* = 0.

Two cohabitation challenge test designs, differing in group structure and family representation, were implemented. Challenge test Design 1 consisted of a few large groups (2 groups with 2000 individuals in each group). All 200 families were represented in all groups, with 10 fish from each family in each group. However, for accurate estimation of genetic infectivity, many small epidemiological groups are required [25, 26]. For that purpose, Challenge test Design 2 consisting of many small groups (20 groups with 200 individuals in each group) were also simulated. Ten different families were distributed across each group, with 20 sibs from each family in each group. In both Challenge Designs, the shedder fish pressure was maintained constant at 2.5%. Thus, the infection was seeded by randomly choosing 50 fish per group as initially infected index cases (IC) in Challenge Design 1 and 5 fish in Challenge Design 2. All remaining fish (1950 and 195 fish per group in Challenge Designs 1 and 2, respectively) were considered to be susceptible.

The epidemic was simulated until no infected individuals remained in the population. In other words, based on the simulated genetic and epidemiological parameters, it was assumed that not all individuals would become infected but that all infected fish would eventually die from the disease. However, to simulate realistic challenge tests, three endpoint criteria were used to define the duration of the test:

- The cumulative mortality reached an asymptote, which occurred at 60 days with 80% of the fish dead
- 50% of the fish per group were dead, which occurred on day 15
- 25% of the fish per group were dead, which occurred on day 7

In the three endpoints scenarios, mortality phenotypes were recorded as time to death (number of days from the start of the challenge test to death). For survivors (either uninfected individuals or infected individuals alive at the end of the test), the phenotype was assigned as one day beyond the final day of the test (i.e., day 61, 16 and 8 for the 80, 50 and 25% mortality designs, respectively). Additionally, for the 50% mortality scenario, an alternative approach was considered in which mortality phenotypes were also recorded as binary outcomes (dead/alive), reflecting a common approach in practice. After selection, mortality percentages were expected to change; however, the endpoint criteria in terms of days were maintained.

### Observed trait

Mortality phenotypes for individuals in each group were obtained as the sum of two components: (i) the infection time (the number of days until an individual becomes infected); and (ii) the infection-induced mortality time (the number of days from infection to death). These components were simulated assuming a stochastic compartmental Susceptible-Infected-Removed epidemiological model [17, 37, 38]. An algorithm adapted from [39], which builds upon Gillespie’s direct algorithm [37], was used to generate inter-event times (i.e., infection time and infection-induced mortality time). These times were determined by individual-level transition rates between compartments: from susceptible to infected (S→I) and from infected to death/removed (I→R). The rate of new infections, ${\lambda}_{j},$ (i.e., the transition from S to I for individual *j*) is governed by the force of infection (summed over all infectious individuals *i* within the same group as individual *j*):

1 $${\lambda}_{j}={\beta \mathrm{e}\mathrm{x}\mathrm{p}(g}_{j}){\sum}_{i}\mathrm{e}\mathrm{x}\mathrm{p}({f}_{i}),$$

where *β* (described previously) is the rate of contacts between susceptible and infected individuals that leads to infection, ${g}_{j}$ is the susceptibility phenotype of individual *j*, and ${f}_{i}$ is the infectivity phenotype of infected individual *i* within the same group. In line with recent empirical evidence [19, 25], the epidemiological model here assumes genetic variation in infection-induced mortality of individuals, which affects the time taken for an infected individual to die (I→R). Therefore, the infection-induced mortality rate is expressed as:

2 $${\gamma}_{j}=\gamma \mathrm{e}\mathrm{x}\mathrm{p}({r}_{j}),$$

where *γ* (described previously) is the average time from infection to death and ${r}_{j}$ describes the infection-induced mortality phenotype of individual *j*.

The time to the next event (either the infection of a susceptible individual, S→ I, or the death of an infected individual, I→R) was sampled from an exponential distribution, with a rate equal to the sum of rates of both processes (infection and infection-induced mortality) (${\mathrm{R}}_{\mathrm{t}\mathrm{o}\mathrm{t}\mathrm{a}\mathrm{l}}=\sum_{j=1}^{n(t)}{\mathrm{r}\mathrm{a}\mathrm{t}\mathrm{e}}_{j}$). The individual chosen to advance to the next specific event was sampled from a uniform distribution weighted by the individual’s infection or infection-induced mortality probability (*p*(*j*) = ${\mathrm{r}\mathrm{a}\mathrm{t}\mathrm{e}}_{j}/{\mathrm{R}}_{\mathrm{t}\mathrm{o}\mathrm{t}\mathrm{a}\mathrm{l}}$) of all alive individuals in the same group. If the chosen individual *j* was susceptible, the next event was ‘infected’ (S → I) and if *j* was infected, then the next event was ‘dead’ (I → R). The simulated epidemic times (infection and infection-induced mortality) were generated until no infected individuals remained in the population.

Importantly, the simulations resulted in time-to-death phenotypes for all infected individuals, regardless of whether death occurred before or after the endpoint criteria used to define the challenge test. This was possible because, as previously noted, simulation continued until no infected individuals remained in the population, regardless of the data collection day, enabling consistent comparison of cumulative mortality at day 60 across the different challenge designs. Nevertheless, the survivor phenotypes used for genetic evaluation were assigned according to the specific endpoint criteria described in challenge test section.

### Genetic evaluation

Genomic evaluations for mortality were performed using the GBLUP method [40, 41, 43]. A linear model was assumed when mortality was recorded as time to death (i.e., continuous trait), whereas a threshold liability model was assumed when mortality was recorded as dead/alive status (i.e., binary trait). In both cases, the model equation was

3 $${\mathbf{y}} = \mu + {\mathbf{Za}} + {\mathbf{e}},$$

where **y** is the vector of observations, µ is the mean, **a** is the vector of random additive genetic effects, **e** is the vector of random residuals, and **Z** is the incidence matrix for genetic effects. Variance–covariance matrices of the random effects were assumed to be **V**(**a**) = **G** ${\sigma}_{{a}_{m}}^{2}$ and **V**(**e**) = **I**_*n*_ ${\sigma}_{{e}_{m}}^{2}$, where **G** is the genomic relationship matrix, computed following VanRaden’s method 1 [44], **I**_*n*_ is the identity matrix of order *n* (number of records), and ${\sigma}_{{a}_{m}}^{2}$ and ${\sigma}_{{e}_{m}}^{2}$ are estimates of the additive genetic and residual variances for mortality, respectively, which were estimated at *t* = 0 for each scenario with different genetic correlations between the underlying traits, using the same model. The average estimates across designs and replicates were ${\sigma}_{{a}_{m}}^{2}$= 60 and ${\sigma}_{{e}_{m}}^{2}$= 250 when mortality was a continuous trait and ${\sigma}_{{a}_{m}}^{2}$= 0.03 and ${\sigma}_{{e}_{m}}^{2}$= 0.16 when mortality was a binary trait.

All individuals, including selection candidates and sibs subjected to the challenge test, were assumed to be genotyped for the 48,000 SNPs (i.e., a panel density of 1600 SNPs/Morgan). This panel was randomly sampled from the 1,020,000 loci with MAF ≥ 0.1 and therefore included only a subset of the causal loci (QTLs) by chance. As noted above, phenotypes (time to death and dead/alive status) were only available for the sibs of the candidates. For a particular generation *t*, the information included in GBLUP (phenotypes and genotypes) was that from the individuals born at *t* and the four preceding generations. The BLUPF90 software [45] was used to carry out the evaluations.

### Selection

Several selection strategies were simulated over 10 discrete generations. First, selection was applied directly on each underlying trait separately. Although this strategy is most effective for reducing disease transmission, it is largely theoretical, as these traits are not directly observable. Second, selection was applied on mortality phenotypes obtained from challenge tests, either time to death or dead/alive status. This approach is more practical and reflects current real-world breeding programs. Table 2 summarizes the simulated schemes that varied according to the observed trait selected and the challenge test design.

**Table 2:** Summary of the schemes simulated that varied in the observed trait selected and in the challenge test design

Trait selected	Challenge test design	No. groups	No. families per group	Duration (days)
Time to death	1	2	200	60
	1	2	200	15
	1	2	200	7
	2	20	10	60
	2	20	10	15
	2	20	10	7
Dead/alive status	1	2	200	15
	2	20	10	15

The latter varied in terms of number of groups, number of families per group and test duration. The percentage of fish dead at the end of test was 80%, 50% and 25% for durations of 60, 15 and 7 days, respectively

When selection targeted the underlying traits, the 100 males and 200 females with the highest true breeding values were chosen to produce the next generation. When selection was based on mortality phenotypes (i.e., time to death or dead/alive status), breeders were chosen based on their estimated breeding values. Then, each male was mated randomly to two females, resulting in 200 full-sib families and 100 half-sib families. Each mating produced 30 fish of which, as indicated above, 10 were designated as selection candidates and the remaining 20 were subjected to challenge testing to obtain the mortality phenotypes.

### Basic reproductive ratio

For each group in the challenge test, the expected theoretical R_0_, assuming that the individual initiating the infection (index case, *IC*)*,* was obtained by extrapolating from [46]:

4 $${\mathrm{R}}_{0}=\left(\beta /\gamma \right)*\left(\sum_{i=1}^{n}{\mathrm{e}\mathrm{x}\mathrm{p}(g}_{i})/n\right)*\left(\mathrm{exp}\left({f}_{IC}\right)/\mathrm{exp}\left({r}_{IC}\right)\right),$$

where *β* and *ϒ* are the mean *per capita* transmission and infection-induced death rates, respectively, as defined above, *n* is the total number of susceptible fish per group (excluding the *IC* fish), ${g}_{i}$ is the susceptibility phenotype of fish *i*, and ${f}_{IC}$ and ${r}_{IC}$ are, respectively, the infectivity and infection-induced mortality phenotypes of the *IC* fish. For each group, a value of R_0_ was obtained for each of the *IC* fish, that is, 50 values per group were obtained in Challenge Design 1 and 5 values per group were obtained in Challenge Design 2. For each generation, R_0_ was summarized as the median R_0_ across the groups instead of the mean, because of the skewed distribution of R_0_.

### Parameters evaluated

Scenarios were compared at each generation based on i) R_0_; ii) the cumulative genetic gain, calculated from the average TBV of each underlying trait; and iii) the cumulative mortality and infected (i.e., number of infected fish) percent at day 60 or later in the challenge test. Results presented are averages over 50 replicates.

## Results

### Direct selection on the underlying traits

**Fig. 2:**
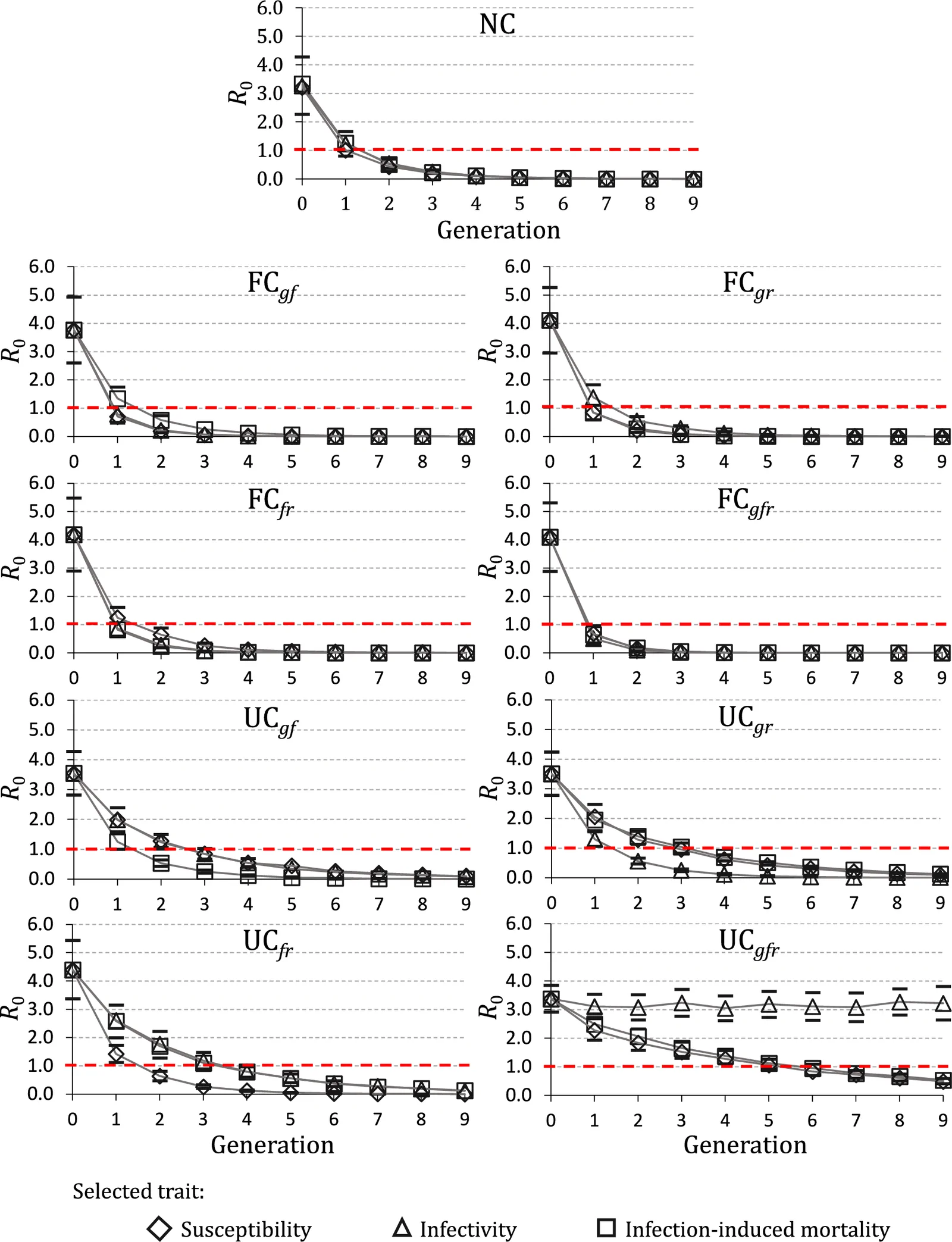
Basic reproductive ratio (R_0_; mean and standard error) across generations according to selected trait and genetic correlations between them. The underlying trait selected was susceptibility (*g*, diamonds), infectivity (*f*, triangles) or infection-induced mortality (*r*, squares). The scenarios considered were NC: underlying traits uncorrelated; FC_*gf*_: favorable positive genetic correlation between *g* and *f*; FC_*fr*_: favorable negative genetic correlation between *f* and *r*; FC_*gr*_: favorable negative genetic correlation between *g* and *r*; FC_*gfr*_: favorable positive genetic correlation between *g* and *f*, favorable negative genetic correlation between *f* and *r* and between *g* and *r*. UC_*gf*_: unfavorable negative genetic correlation between *g* and *f*; UC_*fr*_: unfavorable positive genetic correlation between *f* and *r*; UC_*gr*_: unfavorable positive genetic correlation between *g* and *r*; UC_*gfr*_: unfavorable negative genetic correlation between *g* and *f*, unfavorable positive genetic correlation between *f* and *r* and between *g* and *r*

Applying selection directly to any of the underlying traits represents the optimal theoretical scenario and did not require simulation of a challenge test. In these cases, R_0_ consistently decreased and eventually fell below one for all scenarios with different genetic correlations between traits, except when selecting for infectivity under the least favorable scenario for disease control (UC_*gfr*_; Fig. 2). The generation at which R_0_ dropped below one varied depending on the nature of the genetic correlations between traits.

When the selected trait was uncorrelated with either of the two other underlying traits, the epidemic was eradicated (R_0_ < 1) after just two generations (Fig. 2). Favorable correlations between the selected trait and one (FC_*gf*_, FC_*fr*_ and FC_*gr*_) or both (FC_*gfr*_) of the other underlying traits led to earlier eradication, occurring after a single generation. In contrast, unfavorable correlations (UC_*gf*_, UC_*fr*_. UC_*gr*_ and UC_*gfr*_) delayed eradication, which occurred after three to seven generations (except when selecting for infectivity under UC_*gfr*_, for which the epidemic was never eradicated).

### Selection on mortality recorded as time to death

#### Underlying traits uncorrelated or favorably correlated

**Fig. 3:**
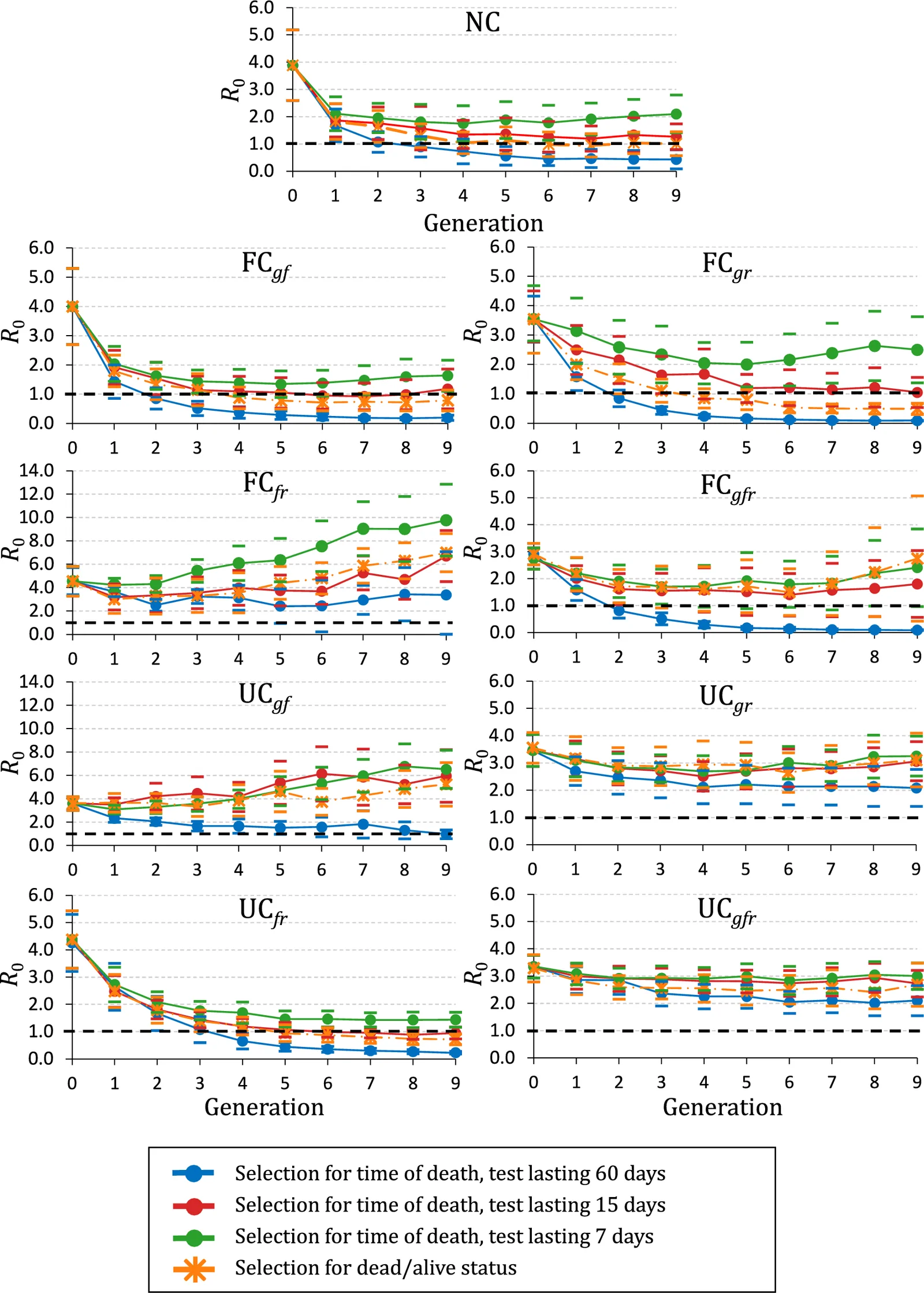
Basic reproductive ratio (R_0_; mean and standard error) across generations when selecting against mortality in challenge design 1. The selected trait was time to death or dead/alive status. The scenarios considered were NC: underlying traits uncorrelated; FC_*gf*_: favorable positive genetic correlation between *g* and *f*; FC_*fr*_: favorable negative genetic correlation between *f* and *r*; FC_*gr*_: favorable negative genetic correlation between *g* and *r*; FC_*gfr*_: favorable positive genetic correlation between *g* and *f*, favorable negative genetic correlation between *f* and *r* and between *g* and *r*. UC_*gf*_: unfavorable negative genetic correlation between *g* and *f*; UC_*fr*_: unfavorable positive genetic correlation between *f* and *r*; UC_*gr*_: unfavorable positive genetic correlation between *g* and *r*; UC_*gfr*_: unfavorable negative genetic correlation between *g* and *f*, unfavorable positive genetic correlation between *f* and *r* and between *g* and *r*

**Fig. 4:**
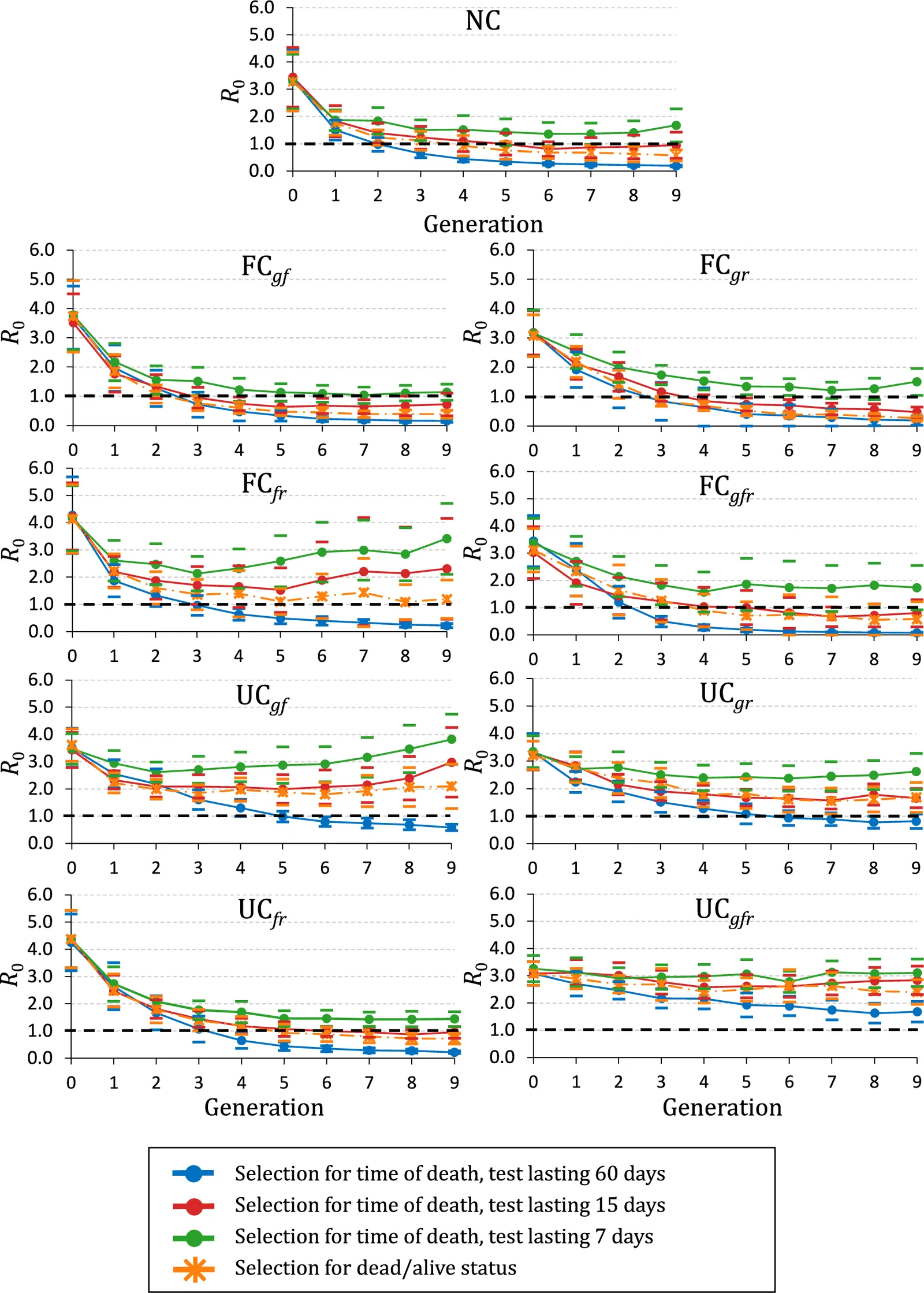
Basic reproductive ratio (R_0_; mean and standard error) across generations when selecting against mortality in challenge design 2. The selected trait was time to death or dead/alive status. The scenarios considered were NC: underlying traits uncorrelated; FC_*gf*_: favorable positive genetic correlation between *g* and *f*; FC_*fr*_: favorable negative genetic correlation between *f* and *r*; FC_*gr*_: favorable negative genetic correlation between *g* and *r*; FC_*gfr*_: favorable positive genetic correlation between *g* and *f*, favorable negative genetic correlation between *f* and *r* and between *g* and *r*. UC_*gf*_: unfavorable negative genetic correlation between *g* and *f*; UC_*fr*_: unfavorable positive genetic correlation between *f* and *r*; UC_*gr*_: unfavorable positive genetic correlation between *g* and *r*; UC_*gfr*_: unfavorable negative genetic correlation between *g* and *f*, unfavorable positive genetic correlation between *f* and *r* and between *g* and *r*

**Fig. 5:**
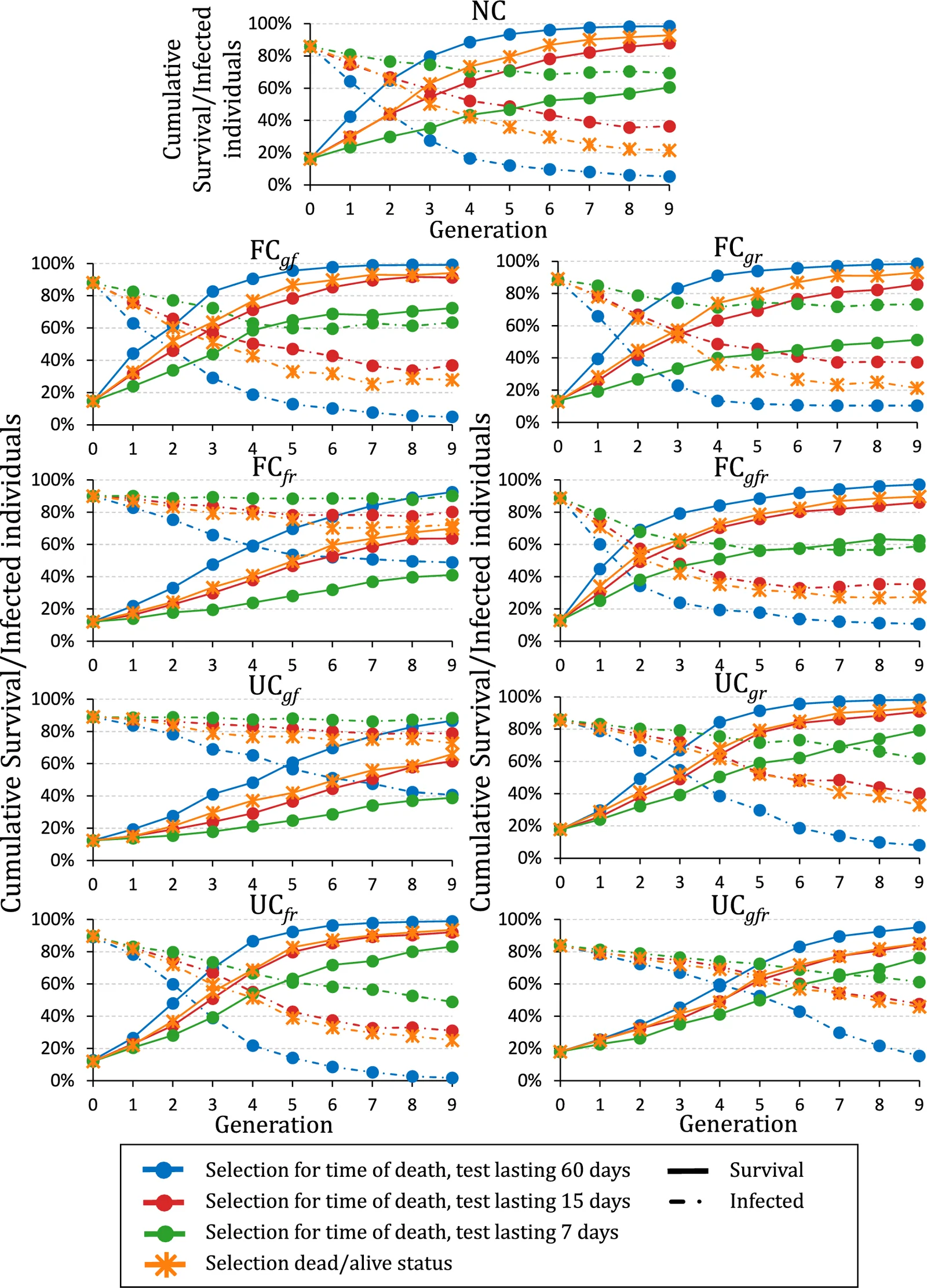
Cumulative survival (solid lines) and infected (dashed lines) percentages at day 60 of the challenge design 1 across generations when selecting against mortality. The selected trait was time to death or dead/alive status. The scenarios considered were NC: underlying traits uncorrelated; FC_*gf*_: favorable positive genetic correlation between *g* and *f*; FC_*fr*_: favorable negative genetic correlation between *f* and *r*; FC_*gr*_: favorable negative genetic correlation between *g* and *r*; FC_*gfr*_: favorable positive genetic correlation between *g* and *f*, favorable negative genetic correlation between *f* and *r* and between *g* and *r*. UC_*gf*_: unfavorable negative genetic correlation between *g* and *f*; UC_*fr*_: unfavorable positive genetic correlation between *f* and *r*; UC_*gr*_: unfavorable positive genetic correlation between *g* and *r*; UC_*gfr*_: unfavorable negative genetic correlation between *g* and *f*, unfavorable positive genetic correlation between *f* and *r* and between *g* and *r*. Standard errors ranged from 0.05 to 0.1

**Fig. 6:**
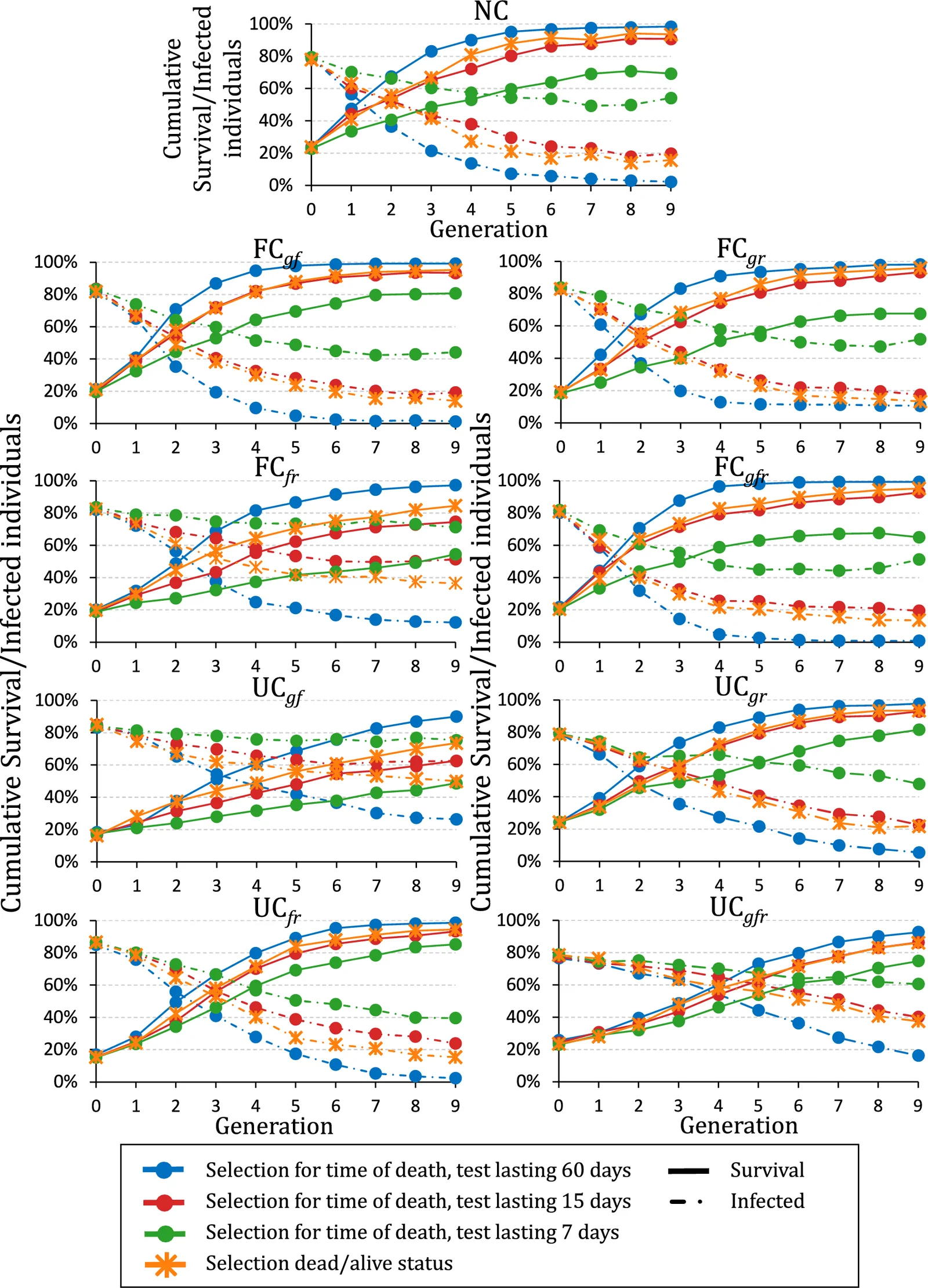
Cumulative survival (solid lines) and infectivity (dashed lines) percentages at day 60 of the challenge design 2 across generations when selecting against mortality. The selected trait was time to death or dead/alive status. The scenarios considered were NC: underlying traits uncorrelated; FC_*gf*_: favorable positive genetic correlation between *g* and *f*; FC_*fr*_: favorable negative genetic correlation between *f* and *r*; FC_*gr*_: favorable negative genetic correlation between *g* and *r*; FC_*gfr*_: favorable positive genetic correlation between *g* and *f*, favorable negative genetic correlation between *f* and *r* and between *g* and *r*. UC_*gf*_: unfavorable negative genetic correlation between *g* and *f*; UC_*fr*_: unfavorable positive genetic correlation between *f* and *r*; UC_*gr*_: unfavorable positive genetic correlation between *g* and *r*; UC_*gfr*_: unfavorable negative genetic correlation between *g* and *f*, unfavorable positive genetic correlation between *f* and *r* and between *g* and *r*. Standard errors ranged from 0.05 to 0.1

When the underlying traits were uncorrelated or favorably correlated, applying selection on time to death led in some designs to epidemic eradication (R_0_ < 1). Under Challenge Design 1, eradication occurred only when the test duration was 60 days in the NC, FC_*gf*_, FC_*gr*_, and FC_*gfr*_ scenarios (blue lines in Fig. 3). Challenge Design 2, with many small groups, produced better outcomes, as eradication was achieved after 60 days for all favorable and uncorrelated scenarios (NC, FC_*gf*_, FC_*gr*_, FC_*fr*_ and FC_*gfr*_; blue lines in Fig. 4), and even when the test lasted 15 days for all FC scenarios, except FC_*fr*_ (red lines in Fig. 4). R_0_ values below 1 were obtained after three to four generations of selection when the test lasted 60 days, and after four to six generations when the test lasted 15 days. In the generation in which R_0_ fell below one, survival rates at day 60 exceeded 80%, while the proportion of infected fish remained below 25% (Figs. 5 and 6).

A notable result was observed in the favorably correlated scenario FC_*fr*_ under Challenge Design 1, where selection for time to death increased R_0_ values to 7.0 and 9.8 when the test lasted 15 and 7 days, respectively (red and green lines in Fig. 3). Despite these elevated R_0_ values, survival rates still improved, and the percentage of infected fish slightly decreased by day 60 when the test lasted 15 days (Fig. 5). However, the percentage of infected fish after 60 days increased, reaching up to 8% (data not shown).

Cumulative genetic gains for the underlying traits at *t* =1 and at *t* = 8, are presented in Tables 3 and 4. For all scenarios, the fish population became less susceptible with each successive generation. The NC scenario showed the highest reduction in susceptibility, with decreases by a factor of 2.7 and 2.2 by *t* = 8 in Challenge Designs 1 and 2, respectively. Additionally, infected fish survived longer after infection with each generation, indicating a decrease in infection-induced mortality (i.e., an increase in endurance). The largest reduction in infection-induced mortality occurred in the FC_*fr*_ scenario, with decreases in *t* = 8 by a factor of 2.5 in Challenge Design 1 (Table 3) and 0.8 in Design 2 (Table 4). It is important to note that, while reduced susceptibility benefits both individual fish and overall epidemic control, reduced infection-induced mortality only benefits the individual. In fact, it may hinder disease control by allowing infected fish to survive longer and continue transmitting the pathogen.

**Table 3:** Cumulative genetic gain for the different underlying traits across generations (*t*) in challenge design 1 when selecting against mortality

	Time to death			Dead/alive status		
*t*	*g*	*f*	*r*	*g*	*f*	*r*
NC scenario						
1	− 0.60	0.00	− 0.19	− 0.49	0.00	− 0.29
8	− 2.73	0.05	− 1.22	− 2.23	0.05	− 1.51
FC_*gf*_ scenario						
1	− 0.57	− 0.28	− 0.19	− 0.46	− 0.22	− 0.28
8	− 2.18	− 1.09	− 1.09	− 1.78	− 0.93	− 1.43
FC_*fr*_ scenario						
1	− 0.51	0.17	− 0.33	− 0.43	0.21	− 0.41
8	− 2.09	1.28	− 2.53	− 1.89	1.39	− 2.73
FC_*gr*_ scenario						
1	− 0.44	− 0.01	0.00	− 0.33	− 0.01	− 0.14
8	− 1.60	− 0.02	− 0.22	− 1.28	− 0.01	− 0.92
FC_*gfr*_ scenario						
1	− 0.52	− 0.15	− 0.12	− 0.40	− 0.05	− 0.25
8	− 1.89	− 0.38	− 0.91	− 1.45	0.12	− 1.67
UC_*gf*_ scenario						
1	− 0.61	0.29	− 0.20	− 0.51	0.25	− 0.32
8	− 3.61	1.70	− 1.73	− 2.80	1.36	− 2.25
UC_*fr*_ scenario						
1	− 0.62	− 0.08	− 0.19	− 0.49	− 0.16	− 0.33
8	− 3.01	− 0.49	− 0.90	− 2.15	− 0.76	− 1.49
UC_*gr*_ scenario						
1	− 0.66	0.00	− 0.44	− 0.60	− 0.01	− 0.50
8	− 3.68	0.04	− 2.92	− 2.82	0.01	− 2.55
UC_*gfr*_ scenario						
1	− 0.65	0.21	− 0.32	− 0.55	0.13	− 0.35
8	− 3.80	1.02	− 2.24	− 2.69	0.49	− 1.90

The selected trait was time to death (test was stopped when mortality naturally levels off) or dead/alive status (test was stopped when 50% of the fish were dead). The underlying traits were susceptibility (*g*), infectivity (*f*) and infection-induced mortality (*r*). Standard errors were equal to 0.1 for *g*, 0.05 for *f*, and 0.25 for *r.* NC: underlying traits uncorrelated; FC_*gf*_: favorable positive genetic correlation between *g* and *f*; FC_*fr*_: favorable negative genetic correlation between *f* and *r*; FC_*gr*_: favorable negative genetic correlation between *g* and *r*; FC_*gfr*_: favorable positive genetic correlation between *g* and *f*, favorable negative genetic correlation between *f* and *r* and between *g* and *r*. UC_*gf*_: unfavorable negative genetic correlation between *g* and *f*; UC_*fr*_: unfavorable positive genetic correlation between *f* and *r*; UC_*gr*_: unfavorable positive genetic correlation between *g* and *r*; UC_*gfr*_: unfavorable negative genetic correlation between *g* and *f*, unfavorable positive genetic correlation between *f* and *r* and between *g* and *r*

**Table 4:** Cumulative genetic gain for the different underlying traits across generations (*t*) in challenge design 2 when selecting against mortality

	Time to death			Dead/alive status		
*t*	*g*	*f*	*r*	*g*	*f*	*r*
NC scenario						
1	− 0.39	− 0.08	− 0.12	− 0.34	− 0.06	− 0.13
8	− 2.18	− 0.31	− 0.67	− 1.95	− 0.24	− 1.10
FC_*gf*_ scenario						
1	− 0.42	− 0.27	− 0.12	− 0.36	− 0.24	− 0.16
8	− 2.08	− 1.24	− 0.51	− 1.72	− 1.04	− 0.91
FC_*fr*_ scenario						
1	− 0.38	0.01	− 0.09	− 0.35	0.06	− 0.22
8	− 1.97	0.24	− 0.83	− 1.98	0.69	− 1.74
FC_*gr*_ scenario						
1	− 0.32	− 0.12	0.04	− 0.22	− 0.10	− 0.06
8	− 1.58	− 0.37	0.13	− 1.22	− 0.40	− 0.45
FC_*gfr*_ scenario						
1	− 0.33	− 0.17	− 0.01	− 0.31	− 0.13	− 0.10
8	− 1.78	− 0.96	− 0.01	− 1.51	− 0.49	− 0.74
UC_*gf*_ scenario						
1	− 0.39	0.13	− 0.08	− 0.38	0.09	− 0.11
8	− 2.69	0.88	− 0.56	− 2.43	0.84	− 1.23
UC_*fr*_ scenario						
1	− 0.47	− 0.14	− 0.15	− 0.39	− 0.16	− 0.22
8	− 2.56	− 0.61	− 0.74	− 2.04	− 0.78	− 1.20
UC_*gr*_ scenario						
1	− 0.49	− 0.10	− 0.24	− 0.42	− 0.10	− 0.29
8	− 2.85	− 0.43	− 1.74	− 2.53	− 0.33	− 2.02
UC_*gfr*_ scenario						
1	− 0.46	0.08	− 0.27	− 0.38	0.04	− 0.26
8	− 3.09	0.64	− 1.76	− 2.33	0.25	− 1.81

The selected trait was time to death (test was stopped when mortality naturally levels off) or dead/alive status (test was stopped when 50% of the fish were dead). The underlying traits were susceptibility (*g*), infectivity (*f*) and infection-induced mortality (*r*). Standard errors were equal to 0.1 for *g*, 0.05 for *f*, and 0.25 for *r*. NC: underlying traits uncorrelated; FC_*gf*_: favorable positive genetic correlation between *g* and *f*; FC_*fr*_: favorable negative genetic correlation between* f* and *r*; FC_*gr*_: favorable negative genetic correlation between *g* and *r*; FC_*gfr*_: favorable positive genetic correlation between *g* and *f*, favorable negative genetic correlation between *f* and *r* and between *g* and *r*. UC_*gf*_: unfavorable negative genetic correlation between *g* and *f*; UC_*fr*_: unfavorable positive genetic correlation between *f* and *r*; UC_*gr*_: unfavorable positive genetic correlation between *g* and *r*; UC_*gfr*_: unfavorable negative genetic correlation between *g* and *f*, unfavorable positive genetic correlation between *f* and *r* and between *g* and *r*

Response in infectivity when selecting on time to death depended on its genetic correlations with the other underlying traits and the challenge test design. When infectivity was genetically uncorrelated with the other underlying traits (i.e., NC and FC_*gr*_ scenarios), no response was observed in Challenge Design 1, while the mean EBV decreased by approximately 0.4 units by *t* = 8 relative to *t* = 0 in Challenge Design 2. When infectivity was positively correlated with susceptibility (FC_*gf*_), selection for time to death led to reductions in the mean EBV for infectivity of 1.1 and 1.2 units by *t* = 8 in Challenge Designs 1 and 2, respectively. In contrast, when infectivity was negatively correlated with infection-induced mortality (FC_*fr*_), infectivity increased over generations, with increases of 1.2 units in Challenge Design 1 and 0.2 units in Design 2 by *t* = 8.

In the most favorable scenario (FC_*gfr*_), time to death selection reduced all three underlying traits in Challenge Design 1. In Challenge Design 2, however, only susceptibility and infectivity were reduced, with no response observed for infection-induced mortality. It is important to note that the most favorable outcome from an epidemic control perspective corresponds to individuals with both low susceptibility and low infectivity but high infection-induced mortality (i.e., low endurance).

#### Underlying traits unfavorably correlated

When the underlying traits were unfavorably correlated, time to death selection reduce R_0_ only during the initial phase of selection in the UC_*gf*_, UC_*gr*_ and UC_*gfr*_ scenarios. However, this reduction was not sustained across subsequent generations, R_0_ either remained stable (UC_*gr*_ and UC_*gfr*_) or even increased (UC_*gf*_) in both Challenge Designs (Figs. 3 and 4). An exception was Challenge Design 2 with a 60-day test duration, where R_0_ continued to decline and eventually fell below one after six and eight generations in, respectively, the UC_*gf*_ and UC_*gr*_ scenarios.

Despite R_0_ increasing or remaining unchanged in UC_*gf*_, UC_*gr*_ and UC_*gfr*_ scenarios, survival rates improved and the percentage of infected fish decreased (Figs. 5 and 6). In the UC_*gr*_ and UC_*gfr*_ scenarios, although the percentage of infected fish at day 60 was reduced, the percentage of infected fish after 60 days increased, reaching up to 45% in Challenge Design 1 and up to 28% in Challenge Design 2 (data not shown). This indicates that, although the number of infected fish was not reduced, the onset of infection was delayed. In contrast, in other unfavorable scenarios and challenge designs for which R_0_ remained greater than one, the percentage of infected fish after day 60 remained below 10% (data not shown).

As in the uncorrelated and favorably correlated scenarios, the fish population became progressively less susceptible with each generation and infected fish survived longer (Tables 3 and 4). The greatest reductions were observed in the UC_*gr*_ and UC_*gfr*_ scenarios, for which by *t* = 8, susceptibility had decreased by a factor of 3.8 in Challenge Design 1 and 3.1 in Challenge Design 2. Similarly, the greatest reduction in infection-induced mortality occurred in the UC_*gr*_ and UC_*gfr*_ scenarios, with decreases of up to 2.9 and 1.8 in Challenge Designs 1 and 2, respectively.

With unfavorable genetic correlations, genetic response in infectivity also depended on its genetic correlations with the other underlying traits and the challenge test design. In the UC_*gr*_ scenario, there was no response in infectivity in Challenge Design 1, while a modest reduction of up to 0.4 was observed in Challenge Design 2. When infectivity was positively correlated with infection-induced mortality (UC_*fr*_ scenario), selection for time to death led to reductions in infectivity over generations by up to 0.5 and 0.6 by *t* = 8 in Challenge Designs 1 and 2, respectively. In contrast, when infectivity was negatively correlated with susceptibility (UC_*gf*_ scenario), infectivity increased over generations, reaching up to 1.7 in Challenge Design 1 and 0.9 in Design 2 by *t* = 8.

At the other extreme, in the least favorable scenario (UC_*gfr*_), time to death selection decreased susceptibility and infection-induced mortality but increased infectivity in both Challenge Designs.

### Selection on mortality recorded as dead/alive status

When selection was based on dead/alive status, the epidemic was only eradicated in the FC_*gf*_, FC_*gr*_ and UC_*fr*_ scenarios in Challenge Design 1 (Fig. 3). Again, Challenge Design 2 produced better outcomes, achieving eradication in the same scenarios as Challenge Design 1, and, additionally, also in the NC and FC_*gfr*_ scenarios (Fig. 4). In the remaining scenarios, R_0_ values remained constant (NC, UC_*gr*_ and UC_*gfr*_ in Challenge Design 1 and FC_*fr*_, UC_*gf*_, UC_*gr*_ and UC_*gfr*_ in Challenge Design (2) or even increased (FC_*fr*_, FC_*gfr*_ and UC_*gf*_ in Challenge Design (1) after the initial generations (Figs. 3 and 4), similar to the pattern observed with time to death selection.

As expected, and consistent with the R_0_ results, survival percentages under dead/alive status selection were always lower than those observed with time to death selection when the test lasted 60 days, but higher when the test lasted 15 days. A similar pattern was observed for the percentage of infected fish: status selection resulted in a higher percentage than time to death selection in the 60-day test, but in a lower percentage in the 15-day test. The percentage of infected fish after 60 days in the UC_*gr*_ scenarios was similar to that found for equivalent scenarios in time to death selection when the test lasted 60 days (data not shown). However, the percentage of infected fish after 60 days in the UC_*gfr*_ scenarios was lower than for time to death selection when the test lasted 60 days for Challenge Design 1 (21 versus 45%, data not shown).

Dead/alive status selection reduced susceptibility in all scenarios, although less than time to death selection when the test lasted 60 days (up to 44 and 33% in Challenge Designs 1 and 2 respectively, Tables 3 and 4). Status selection also reduced infection-induced mortality in all scenarios, although more than time to death selection when the test lasted 60 days (up to 76% and 130% in Challenge Designs 1 and 2 respectively). Responses in infectivity, followed the same trends for dead/alive status selection and time to death selection, with increasing or decreasing infectivity for the corresponding scenarios.

In the most favorable scenario (FC_*gfr*_), selection based on dead/alive status resulted in a reduction of the three underlying traits in Challenge Design 2. In contrast, in Challenge Design 1 and in the least favorable scenario (UC_*gfr*_) in both Challenge Designs, dead/alive selection decreased susceptibility and infection-induced mortality but increased infectivity.

## Discussion

In this study, a severe outbreak in an aquaculture population was simulated to evaluate the effectiveness of genomic selection for different traits in controlling disease transmission. The results demonstrated that direct selection on true breeding values for the underlying traits was highly effective, with the epidemic being eradicated (R_0_ < 1) in most scenarios evaluated after just two generations of selection. However, to our knowledge, there are currently no commercial breeding programs that directly select for underlying epidemiological traits.

Selection on mortality (time to death or status) generally led to reductions in both mortality and R_0_ over successive generations. While these reductions were consistent across scenarios, the rate of improvement declined over generations, partly due to decreasing prediction accuracy when the number of infected or dead individuals was low. Nevertheless, as outlined by Bijma et al. [14], disease eradication can still be achieved despite these diminishing prediction accuracies. When selection targeted the observed trait time to death and the challenge test lasted 60 days (i.e., until mortality naturally plateaued), R_0_ values below one were achieved after three to four generations of selection. In contrast, selection on dead/alive status was generally ineffective in preventing disease spread.

Regardless of whether mortality was recorded as time to death or as dead/alive status, selection against mortality consistently reduced both susceptibility and infection-induced mortality (i.e., increased endurance). However, the response in infectivity varied depending on the genetic correlations between this trait and the other underlying traits.

It is important to note that, in commercial aquaculture breeding programs, disease resistance is typically included as part of a multi-trait selection index, alongside production traits such as growth. Consequently, the number of generations required to effectively control disease transmission in practice will exceed those observed in our simulations, where disease resistance was given full priority.

### Impact of selecting against mortality on the underlying traits

Fundamentally, selecting against mortality (whether based on time of death or alive/dead status) results in selecting survivors. One reason an individual may survive is its low susceptibility, which enables it to avoid infection. This explains the observed reduction in susceptibility across all scenarios, regardless of the genetic correlations with the other underlying traits.

The reduction in susceptibility from selection on time to death was more pronounced with longer challenge tests. For a given infection pressure, individuals with high susceptibility tend to become infected first, followed by those with medium and finally with low susceptibility. Therefore, shorter challenge tests result in fewer infected individuals, primarily those with high susceptibility. In contrast, longer tests (e.g., until mortality naturally levels off), allow sufficient time for infection to spread to individuals with medium susceptibility as well, ensuring that nearly all non-resistant individuals are exposed.

In principle, as suggested in previous studies [13, 14, 47], a reduction in susceptibility can lead to a decrease in R_0_ through two main mechanisms: i) fish with low susceptibility are less likely to become infected (i.e., less infected fish in the group); and ii) for a fixed challenge test duration, these fish spend less time in an infectious state, thereby reducing their opportunity to transmit the disease to others. However, our results show that despite a reduction in susceptibility, selection against mortality did not always translate into a reduction in R_0_ and the extent of R_0_ reduction depended on the genetic correlations between the underlying traits. For example, in the FC_*fr*_ scenario (with a positive genetic correlation between infectivity and infection-induced mortality) and in the UC_*gf*_ scenario (with a negative genetic correlation between susceptibility and infectivity), R_0_ did not decrease after the first two generations. In these scenarios, although susceptibility decreased, infectivity increased. As a result, fish became less susceptible but more infective over generations. At a phenotypic level (observed during the challenge test), this manifests as a higher survival rate over time (under a fixed 60 day test duration) but only a slight decrease in the percentage of infected fish. Consequently, pathogen transmission was not effectively reduced.

Individuals may also survive because of low infection-induced mortality, which allows them to remain alive even after becoming infected. Notably, the proportion of survivors attributed to low infection-induced mortality is expected to decrease with longer challenge test durations, as infected fish are eventually given enough time to succumb to the disease. As a result, selection for time to death at 60 days leads to a smaller reduction in genetic gain for infection-induced mortality compared to selection at earlier time points, such as 15 days (based on time to death or dead/alive status) or 7 days (based solely on time to death).

Although reduced infection-induced mortality contributes positively to overall survival rates, it can have a negative effect on R_0_, as fish that survive longer while remaining infectious have more opportunities to transmit the pathogen to others, thereby increasing the potential for disease transmission. Thus, while selecting against mortality improves survival, it may sustain or even increase R_0_, unless the accompanying reduction in infectivity or susceptibility are sufficient to counterbalance the prolonged infectious period. This dynamic was observed in the UC_*gr*_ and UC_*gfr*_ scenarios under both Challenge test Designs, and in the FC_*fr*_ scenario under Challenge Design 1.

Finally, another reason individuals may survive is due to low viral load in their environment, which results from low infectivity within the group. As described in various studies [14, 48, 49], infectivity is a complex trait because it is not host-dependent; that is, whether an individual becomes infected depends on the infectivity of others in its group, not on its own capacity to transmit the disease. Thus, in our simulations, responses in infectivity were always indirect, driven by either genetic correlations with other traits or by the design of the challenge test. When infectivity was genetically uncorrelated with the other underlying traits and all families were evenly mixed within groups (as in Challenge Design 1), no response in infectivity was observed when selecting against mortality. However, when unfavorable correlations existed between infectivity and susceptibility or between infectivity and infection-induced mortality, selection against mortality could lead to inadvertent increases in infectivity. Conversely, favorable genetic correlations among underlying traits facilitated a reduction in infectivity as a correlated response. In practical breeding programs, such unfavorable genetic correlations could undermine disease control, as selecting for reduced mortality might unintentionally promote higher infectivity in the population. Careful consideration of these genetic correlations is therefore important to avoid compromising disease control.

### Challenge test designs

The impact of challenge test design on disease transmission was assessed by varying the number of groups, group sizes, and the distribution of families across these groups. Challenge Design 1 simulated a simple structure with two large groups of 2000 fish each. Families were evenly mixed within these groups. Although this design does not allow for distinguishing infectivity differences between groups, it was prioritized in the results due to its frequent use in practice, primarily because of logistical constraints such as limited tank space for challenge testing [20, 22–24]. To explore the effect of group-specific infectivity, Challenge Design 2 involved 20 smaller groups, each with 200 fish. In this setup, each group contained 10 families, with family composition varying across groups. This design mirrors challenge test setups used in studies such as [36] on Atlantic Salmon and ISAV. A third design also involved 20 small groups (200 fish each), but with families equally mixed across all groups. Since the outcomes of this design closely matched those of Challenge Design 1, its results were not presented.

The main differences observed between Challenge Designs 1 and 2 related to infectivity, which was influenced by the distribution of families across groups. In Challenge Design 1, all families were represented in both groups, equalizing mean infectivity and reducing between-group variation in disease dynamics. In contrast, Challenge Design 2 assigned ten distinct families to each group, creating differences in group-level infectivity. Groups composed primarily of low-infectivity families often showed limited or no epidemic spread, resulting in more survivors, whereas groups dominated by high-infectivity families experienced faster disease transmission, resulting in earlier infections and, therefore, higher overall mortality. This between-group variation enabled the identification of families with extreme infectivity values, generating a detectable selection response for infectivity—even in the absence of genetic correlations with other traits (NC scenario). The modest reduction in infectivity observed under Challenge Design 2 led to lower infection prevalence and reduced R_0_ values compared to Challenge Design 1.

It is also important to note that selection based on dead/alive status was found to be more sensitive to the challenge test design than selection based on time to death at 60 days. In contrast to Challenge Design 1, in Challenge Design 2, dead/alive selection resulted in R_0_ values below 1 in more scenarios (e.g., NC and FC_*gfr*_), and did not increase R_0_ in scenarios such as FC_*fr*_ and UC_*gf*_. These findings suggest that increasing the number of groups and diversifying family representation in them offers better cost-benefits ratios when selection is based on dead/alive status.

### Impact of selecting for time to death versus dead/alive status on R_0_

As previously discussed, disease resistance in aquaculture selection programs is typically assessed using mortality data [5, 7–9]. Mortality is generally recorded either as time to death, measured until mortality naturally levels off, or as a binary survival status (dead/alive) when cumulative mortality reaches approximately 50% [18, 28, 38]. In practice, the binary approach is more commonly adopted [20, 24, 36, 50–55], primarily because it is simpler, requires a shorter challenge period (e.g., ~ 14 days versus 60 days in ISAV challenges to reach 50% mortality), and is more cost-effective compared to recording time to death.

In this study, selection based on time to death consistently outperformed selection based on dead/alive status across all scenarios considered. Differences in survivor profiles described previously explain the greater reduction in susceptibility and lower reduction in infection-induced mortality, and, therefore, in R_0_ with selection on time to death. When selection was based on time to death, fish with low susceptibility were primarily selected, while when selection was based on dead/alive status, fish that were both less susceptible and less likely to die from infection (i.e., with high endurance) were selected.

Notably, these differences in survivor profiles were not reflected in conventional phenotypic measures, such as overall survival percentage, which were similar for both selection approaches. This distinction is particularly important in contexts where dead/alive status selection may delay the onset of infection without significantly reducing outbreak frequency, though it can improve survival at specific time points. For example, when susceptibility and infection-induced mortality are positively genetically correlated (i.e., fish with low susceptibility are also the last to die when infected), dead/alive selection may favor individuals that are not necessarily the most resistant but that are better able to tolerate or delay disease progression. Delaying infection onset or increasing tolerance can be beneficial, particularly for diseases that affect adult fish or those nearing market size, or potentially also when R_0_ values are very high, so that reduction of R_0_ to below one is out of reach. However, this strategy may be less appropriate for diseases impacting juveniles, as it could lead to long-term drawbacks, such as increased vulnerability to other infections or reduced performance later in life.

### Assumptions and limitations of the study

A key requirement for generating informative data to support breeding for reduced disease transmission is that the disease challenge test allows for natural disease transmission within epidemic groups. In our simulations, this was achieved by introducing infected seeder fish (ICs) into groups consisting of initially uninfected cohabitants. Such cohabitation trials are relatively common in aquaculture [19, 20, 36, 56, 57]. However, many disease challenge research experiments instead rely on direct injection or inoculation of pathogens into all individuals [53, 58–60]. By bypassing natural transmission pathways, such experiments do not provide informative data for breeding to reduce disease spread. However, given the common practice of injection or bath challenge studies in aquaculture research, it would be interesting to explore whether selection based on observed mortality records from these trials also leads to higher survival rates and lower disease transmission in natural outbreaks and cohabitation challenge tests.

Our simulations assumed an SIR epidemiological model, a framework commonly used to represent the transmission dynamics of infections that cause mortality or induced long-lasting immunity [16]. An important assumption of this model is that the pathogen replicates only within the host, such that a reduction in the number of infected individuals directly translates into reduced exposure for the population. Hence, our results may not extend to infections involving parasites that have part of their life cycle outside the host (e.g., sea-lice infections). For diseases that follow a Susceptible-Infected-Susceptible (SIS) epidemiological model, where recovery and reinfection can occur, selection outcomes may differ from those expected under SIR framework, as disease eradication is likely more challenging and reducing susceptibility alone may be less effective, while other traits such as infectivity, duration of infection, and recovery rate become more relevant.

In this study, due to limited empirical information and in order to simplify the simulated scenarios, identical phenotypic and genetic variances were assumed for the three underlying epidemiological traits. Differences in the magnitude of phenotypic and genetic variances between underlying traits are expected to influence both the relative contribution of susceptibility and endurance to observed survival phenotypes, as well as R_0_ and the shape of the transmission curve.

High transmission and mortality rates were assumed in this study, reflecting conditions often achieved in aquaculture experiments through high challenge doses administered to sufficient numbers of seeder fish. Challenge experiments with lower transmission or mortality rates would likely result in slower genetic progress but would not be expected to alter the main conclusions of this study.

Finally, our simulations assumed pathogen transmissibility and virulence do not change over successive generations of selection. However, like all interventions that aim for disease reduction, genetic selection may exert selection pressure on pathogen populations. To date, knowledge about the impact of selection for disease resistance in aquaculture on pathogen evolution is sparse [9]. However, lower rates of gain in R_0_ or survival than predicted in our simulations would be expected if the pathogen was able to adapt [61].

### Future prospects—selection for underlying epidemiological traits affecting R_0_

Recent studies emphasize that interventions to prevent transmission should aim to reduce R_0_ < 1 as quickly as possible, in order to prevent the pathogen from adapting [47, 61]. In our simulation, direct selection on the underlying traits was the most effective strategy, eradicating the disease in just one or two generations—considerably faster than selection based on observed traits. However, it is important to note that our simulations assumed that breeding values for these traits could be predicted with 100% accuracy, which is not be realistic in practical breeding programs.

Furthermore, our simulation results highlight that the response to selection on mortality traits is strongly influenced by the genetic correlations between the underlying epidemiological traits. If genetic correlations were favorable and higher than simulated here, faster reduction in disease prevalence, mortality, and R_0_ would be expected, whereas lower correlations would likely result in poorer outcomes. However, empirical knowledge of these correlations is currently limited and varies across studies. For example, an unfavorable genetic correlation between susceptibility and infection-induced mortality (i.e., less susceptible fish died later) was observed for ISAV in Atlantic salmon [20]. Similarly, for mastitis in cattle, a favorable genetic correlation was found between susceptibility and recoverability (i.e., less susceptible generally recover faster) [62, 63]. In Atlantic salmon, a genetic locus conferring high resistance (i.e. 70% lower mortality rates in challenge test) to Infectious Pancreatic Necrosis was associated with low susceptibility, low infectivity, and high infection-induced mortality, representing an optimal combination for reducing disease transmission [64]. In common carp, a favorable genetic correlation between susceptibility and infectivity has been reported for Koi herpesvirus [49]. A similar favorable genetic correlation has also been observed for bovine leukemia virus [65]. In contrast, no clear genetic correlation between susceptibility and infectivity has been reported for bovine Tuberculosis [66], and a low unfavorable genetic correlation has been observed for digital dermatitis [67] in cattle.

Despite their importance in genetic disease control, measuring or estimating the epidemiological host traits and their genetic correlations remains challenging. To our knowledge, no commercial breeding programs currently estimate or directly select for these underlying traits. Recent work has addressed this limitation through the development of statistical-genetics tools to estimate genomic breeding values for R_0_ [14, 34], as well as methods that provide simultaneous estimates of SNP effects or genetic parameters for all three underlying epidemiological traits [26, 42] and guidelines for designing disease challenge test aimed at quantifying these epidemiological traits [19, 25, 27, 49]. Importantly, theoretical predictions and simulations suggest that the experimental design criteria for obtaining unbiased, accurate estimates for the genetic effects (breeding values) for the epidemiological traits coincide with the criteria for achieving the fasted reduction in disease transmission and mortality found in this study: many smaller groups with targeted family composition rather than randomly distributed individuals [26, 27, 42]. These are promising prospects for future aquaculture breeding programs that aim to reduce both pathogen transmission and mortality.

## Conclusions

Our simulation results indicate that genomic selection against disease mortality, when evaluated as time to death in a cohabitation or bath challenge test conducted until mortality naturally levels off, not only reduces mortality but also decreases disease transmission. However, reductions in transmission were not guaranteed, as the correlated responses in susceptibility, infectivity, and infection-induced mortality depended on their underlying genetic correlations.

Selection based on time to death was more effective than selection based on dead/alive status, highlighting the importance of the mortality phenotype used for genetic evaluation. In addition, challenge test designs with multiple epidemic groups and appropriate family structures improved the ability to capture genetic variation affecting disease transmission. Future breeding programs may benefit from challenge test designs and genomic tools that allow estimation of the underlying host traits affecting both disease transmission and survival.

## Data Availability

The Fortran F90 codes are available from the corresponding author on reasonable request.
